# Association Between Sarcopenia and Chronic Renal Failure (Overt and Concealed) in Chronic Obstructive Pulmonary Disease (COPD) Patients: A Cross-Sectional Study

**DOI:** 10.7759/cureus.46870

**Published:** 2023-10-11

**Authors:** Yogesh M, Anjali K Dave, Shubham S Patel, Ram Parbat, Viral Shah, Rohankumar Gandhi

**Affiliations:** 1 Community Medicine, Shri M. P. Shah Government Medical College, Jamnagar, IND; 2 Community and Family Medicine, Shri M. P. Shah Government Medical College, Jamnagar, IND

**Keywords:** : association, observational cross-sectional study, : sarcopenia, chronic renal failure, copd: chronic obstructive pulmonary disease

## Abstract

Background

Sarcopenia, a syndrome characterized by a progressive decline in skeletal muscle mass, strength, and function, is frequently associated with chronic diseases such as chronic obstructive pulmonary disease (COPD). Chronic kidney disease (CKD) is a prevalent condition among patients with sarcopenia. Reports suggest that between 15% and 55% of stable COPD patients have sarcopenia. Therefore, the present study aims to determine the association between sarcopenia and chronic renal failure (overt and concealed) in COPD patients.

Methodology

This institutional-based cross-sectional study was conducted on patients diagnosed with COPD. Hospitalized adult COPD patients who gave consent were included. Sociodemographic information such as age, gender, residence, and prolonged length of stay in the hospital (categorized by a median of 10 days, considering its data distribution in our sample) was obtained using electronic medical records. Skeletal muscle %, visceral fat %, and body fat % were calculated using a bio-electrical impedance analysis device (Omron Body Composition Monitor, Model HBF-702T). Additionally, the strength of the hand grip was measured using a hand dynamometer. Sarcopenia was assessed following the criteria set by the Asian Working Group on Sarcopenia (AWGS). Chronic renal failure (CRF) was assessed by calculating the glomerular filtration rate (GFR) using the Modification of Diet in Renal Disease (MDRD) Study Group equation. Quantitative data were compared using an independent sample t-test. The association was determined using chi-square and multivariate logistic regression analyses. A p-value of <0.05 was considered significant.

Results

The study found that the proportion of sarcopenia in COPD patients was 52%, with overt and concealed CRF prevalence rates of 31.5% and 27%, respectively. Sarcopenic individuals had significantly lower FEV1 and FEV1/FVC compared to non-sarcopenic patients. The incidence of sarcopenia significantly increased with rising BODE index (body mass index (BMI, B), airflow obstruction (O) as measured by the post-bronchodilator FEV_1_ (percentage of predicted value), dyspnea (D) assessed by the modified Medical Research Council (MMRC) score, and exercise tolerance (E) measured by 6-minute walking distance) and mMRC (modified Medical Research Council dyspnea scale) dyspnea scale scores. Both concealed CRF and overt CRF patients had four times higher odds of having sarcopenia (AOR=4).

Conclusion

The study reveals a high prevalence of sarcopenia and provides evidence for the association between sarcopenia and chronic renal failure in COPD patients. These findings underscore the importance of early detection and management of sarcopenia and CRF in COPD patients to optimize their clinical outcomes.

## Introduction

Exposure to noxious particles or gases, particularly tobacco smoke, is a common cause of the progressive respiratory ailment known as chronic obstructive pulmonary disease (COPD), characterized by persistent airflow restriction. COPD is associated with various extrapulmonary manifestations, contributing to its complexity as a multisystem disorder. Among these manifestations, muscle dysfunction, particularly sarcopenia, has gained significant attention due to its profound impact on patients' health outcomes and quality of life [1].

The bidirectional relationship between COPD and sarcopenia stems from overlapping pathological mechanisms. Systemic inflammation, oxidative stress, and altered protein metabolism are common denominators that drive both conditions. Examples of inflammatory mediators elevated in both sarcopenia and COPD include TNF-alpha and interleukins. These molecules contribute to muscle wasting and have adverse effects on renal function, promoting glomerular injury and impairing tubular reabsorption [1].

Chronic conditions like COPD are often linked to sarcopenia, a syndrome marked by a gradual loss in skeletal muscle mass, strength, and function [1]. Airflow restriction and comorbidities are common features of the chronic respiratory disease COPD [2]. With considerable overlap in clinical characteristics and therapies, sarcopenia and COPD are increasingly recognized as related [1].

Several clinical outcomes, including exercise capacity, balance, strength, gait speed, physical activity levels, and health-related quality of life, have been shown to deteriorate when sarcopenia is present in COPD patients. Exacerbations of COPD can also lead to a rapid decrease in muscle mass and functionality [2]. Therefore, understanding the relationship between sarcopenia and COPD is essential for monitoring and treating COPD patients effectively.

Reports suggest that between 15% and 55% of stable COPD patients have sarcopenia [2]. A Korean study reported a frequency of 25% [1]. Sarcopenia is associated with higher risks of unfavorable health outcomes in COPD patients, including death, hospital admission, falls, physical impairment, and poor quality of life [2]. It also shares a close relationship with other diseases, such as frailty syndrome and osteoporosis [3].

In the context of chronic renal failure, fluid and electrolyte imbalances can exacerbate muscle wasting and weaken respiratory muscles, further compromising pulmonary function in COPD patients. Additionally, alterations in mineral metabolism, such as elevated levels of parathyroid hormone (PTH) and phosphorus, are commonly observed in chronic renal failure and have been associated with muscle weakness and reduced exercise capacity. These metabolic disturbances could synergize with COPD-related muscle dysfunction, creating a cycle of progressive decline in both pulmonary and muscular function [4].

Furthermore, there is evidence to suggest that sarcopenia is also associated with chronic renal failure. Chronic kidney disease (CKD) is a prevalent condition among patients with sarcopenia [4]. The relationship between sarcopenia, COPD, and chronic renal failure in COPD patients has not been extensively studied. Therefore, this cross-sectional study aims to determine the association between sarcopenia and chronic renal failure (overt and concealed) in COPD patients.

## Materials and methods

This was an institutional-based cross-sectional study conducted on patients diagnosed with COPD according to the Global Initiative for Chronic Obstructive Lung Disease (GOLD) guidelines. The study took place at the Pulmonary Medicine Department in Gujarat between February 2023 and July 2023. Using the formula 3.84 * PQ/L2, the prevalence of sarcopenia in COPD was estimated at 52% [5].

Based on this calculation (3.84 * 52 * 48/100 = 95), considering a 10% non-responsive rate, the total sample size was determined to be 110.

Adult hospitalized COPD patients were selected randomly by a simple random technique after assessing eligibility criteria, which included being adult hospitalized COPD patients who provided informed, written consent to participate in the study. Participants with active malignancies, contraindications to body composition analysis using bioimpedance methods (such as those with metal implants, implanted cardiac devices, or edemas), those who were critically ill or unable to respond, and patients with risk factors for chronic renal failure (hypertension, diabetes, nephrotoxic drugs, pregnancy, advanced liver disease, and known cardiovascular disease) were excluded from the study.

Data collection tool: Sociodemographic information, including age, gender, residence, and length of stay in the hospital (categorized based on a median of 10 days considering its data distribution in our sample), was obtained using electronic medical records. Anthropometric measurements, such as BMI and WC, were also recorded. Bioelectrical impedance analysis (BIA) was performed using an Omron Body Composition Monitor (Model HBF-702T) to calculate skeletal muscle %, visceral fat %, and body fat %. Handgrip strength was measured using a hand dynamometer.

Diagnosis of sarcopenia: To diagnose sarcopenia, the following measurements were taken:

Muscle mass was evaluated using BIA. Sarcopenia can be diagnosed with BIA if the skeletal muscle mass index (SMMI) is less than 7.0 kg/m2 for males and less than 5.5 kg/m2 for females, following the Asian Working Group of Sarcopenia (AWGS) recommendations for cut-off thresholds for poor muscle mass and strength [6].

Handgrip strength was used to assess muscle strength. The standard cut-off value for diagnosing sarcopenia is a handgrip strength of less than 20 kg for females and less than 27 kg for males, although cut-off values may vary based on factors including age, sex, and the population under study [6].

Dyspnea scales: The modified British Medical Research Council (mMRC) scale was used to assign dyspnea scores ranging from 0 to 4 to individuals [7].

BODE index: The BODE index was calculated as the total of scores obtained from the assessment of four factors: BMI (B), airflow obstruction (O), dyspnea (D), and exercise capacity (E) [8].

Pulmonary function test (PFT): Pulmonary function tests were conducted at the Respiratory Function Laboratory of the Chest Diseases using ATS/ERS-compliant equipment (EasyOne Pro® LAB, Medical Technologies, Inc., Andover, MA, USA).

Diagnosis of chronic renal failure (CRF): The MDRD study group equation was used to calculate the GFR: 170 * [serum creatinine] - 0.999 * [age] - 0.176 * [blood urea] - 0.170 * [serum albumin] 0.318 (0.762 for women) (1.180 for African-American individuals) [9]. Patients were classified as having normal renal function (GFR ≥ 60 mL/min/1.73 m2), concealed CRF (normal serum creatinine and GFR < 60 mL/min/1.73 m2), or overt CRF (increased serum creatinine and GFR < 60 mL/min/1.73 m2). Threshold values for men's serum creatinine were 1.26 mg/dL, and for women, they were 1.04 mg/dL.

Statistical analysis: IBM Corp. Released 2019. IBM SPSS Statistics for Windows, Version 26.0. Armonk, NY: IBM Corp was used for statistical analysis. Descriptive statistics for continuous variables included mean and standard deviation (SD), while categorical data were presented as frequency and percentage (%). Independent sample t-tests were used for numeric data, and chi-squared tests were used for categorical data. Bivariate and multivariate regression analyses were employed to assess the impact of sarcopenia-related parameters and their relation to CRF. A significance threshold of p < 0.05 was used for all results with a 95% confidence interval.

## Results

Table 1 shows the socio-demographic characteristics, length of stay of the study participants, and its association with sarcopenia. Age group greater than 60 years, urban residence, and prolonged hospital stay (>10 days) were associated with statistically significant sarcopenia (p-value<0.05).

**Table 1:** Socio-demographic characteristics and length of stay in the hospital of the study participants p-value<0.05: significant, p-value<0.001: highly significant

Variable	Non-Sarcopenia patients (n=53)	Sarcopenia patients (n=58)	OR (CI)	p-value
Age				
>60 years	16(33)	32(67)	2.84 (1.30-6.22)	0.008*
<60 years	37(59)	26(41)		
Gender				
Female	33(54)	28(46)	1.76 (0.82-3.7)	0.140
Male	20(40)	30(60)		
Residence				
Urban	20(32)	42(68)	4.33 (1.94-9.6)	0.003*
Rural	33(67)	16(33)		
Length of Hospital Stay				
Normal	31(78)	9(22)	7.67 (3.2-18.8)	<0.001**
Prolonged	22(31)	49(69)		

Table 2 shows the comparison of anthropometric and bio-impedance parameters of the study participants (sarcopenic vs. non-sarcopenic patients). The mean age was higher in sarcopenia patients than in non-sarcopenia patients, which is statistically significant. In anthropometric characteristics, weight, BMI, and WC were lower compared with non-sarcopenic patients, which is statistically significant. In bio-impedance indices, muscle mass (in kg), body fat %, visceral fat %, skeletal muscle %, and handgrip were lower in sarcopenia compared to non-sarcopenic patients, which is statistically significant.

**Table 2:** Comparison of anthropometric and bio-impedance parameters of the study participants p-value<0.05: significant, p-value<0.001: highly significant

Variable	Non-Sarcopenia patients (n=53), Mean ± SD	Sarcopenia patients (n=58), Mean ± SD	t-value	p-value
Age	52 ± 14	58 ± 16	2.094	0.03*
WC	112 ± 16.2	92.6 ± 12.3	-7.165	<0.001**
Weight	56 ± 12.6	46± 14.9	-3.799	0.002*
BMI	22.6 ± 4.1	18.4 ± 5.2	-4.695	<0.001**
Muscle mass(kg)	52 ± 6.2	41 ± 5.2	-10.157	<0.001**
BF%	28.2 ± 9.2	24 ± 10.1	-2.283	0.02*
VF%	14.9 ± 10.6	10.2 ± 5.8	-2.931	0.04*
SM%	19.98 ± 4.19	5.9 ± 1.2	-24.52	<0.001**
Handgrip	13.13 ± 7.2	8 ± 6.8	-3.86	0.002*

Table 3 shows the impact of sarcopenia on the pulmonary function test, mMRC, and BODE index of COPD patients. The percentages of FEV1 and FEV1/FVC were significantly lower in the sarcopenic patients compared to the nonsarcopenic patients. As the BODE index and mMRC dyspnea scale scores increased, the incidence of sarcopenia increased significantly.

**Table 3:** Impact of sarcopenia on the COPD severity of the patients p-value<0.05: significant, p-value<0.001: highly significant

Variables	Non-Sarcopenia patients (n=53), Mean ± SD	Sarcopenia patients (n=58), Mean ± SD	t-value	p-value
FEV1%	45.6 ±16.5	40 ± 14.4	-1.90	0.04*
FVC%	56 ±18.8	51.1 ± 17.6	-1.41	0.15
FEV1/FVC	61.1 ± 7.2	58.1 ± 6.2	-2.3	0.02*
mMRC scores	1.62 ±1.4	2.5 ± 1.2	3.56	0.005*
BODE index	3.9± 2.9	5.6 ± 2.8	3.14	0.002*

Table 4 shows the impact of sarcopenia on the laboratory investigations of the patients. Serum hemoglobin, GFR, and total protein were lower in sarcopenic patients than in non-sarcopenic patients. Serum creatinine levels are higher in sarcopenic patients than in non-sarcopenic patients, which is statistically significant.

**Table 4:** Impact of sarcopenia on the laboratory investigation of the patients p-value<0.05: significant, p-value<0.001: highly significant

Variables	Non-Sarcopenia patients (n=53), Mean ± SD	Sarcopenia patients (n=58), Mean ± SD	t-value	p-value
Hb	12.93 ± 1.24	11.6 ± 1.04	-6.14	<0.001**
ESR	33.9 ± 22.74	35.6 ± 31.28	0.325	0.74
Total Protein	7.07 ± 0.37	6.09 ± 0.35	-14.33	<0.001**
Creatinine	1.22 ± 0.39	2.11 ± 0.51	10.25	<0.001**
Urea	40.5 ± 26.1	44.5 ± 31.3	0.72	0.46
GFR	93.05 ± 25.64	80.66 ± 34.9	-2.11	0.03*

Table 5 shows the association between chronic renal failure (overt and concealed) and sarcopenia in multivariate logistic regression. Both concealed CRF and overt CRF patients have four times higher odds of having sarcopenia than those who don’t have CRF.

**Table 5:** Association between chronic renal failure (overt + concealed) and sarcopenia p-value<0.05: Significant, p-value<0.001: Highly significant, AOR: Adjusted odds ratio

Variables	Non-Sarcopenia patients (n=53)	Sarcopenia patients (n=58)	AOR (CI)	p-value
Concealed CRF				
Present	7(23)	23(77)	4.11 (1.52 – 10.20)	0.0026*
Absent	46(57)	35(43)		
Overt CRF				
Present	9(26)	26(74)	3.80 (1.64 – 8.61)	0.0022*
Absent	44(58)	32(42)		

## Discussion

The present study found that the prevalence of sarcopenia in COPD patients was 52%, with the prevalence of overt and concealed CRF at 31.5% and 27%, respectively. Previous studies have shown that patients with COPD had a frequency of overt CRF of 6.62% and concealed CRF of 19.85% [9]. The prevalence of sarcopenia in COPD males over the age of 40 varies from 20% to 40%, depending on the population's age, gender, and other variables [10]. According to the study by Kon SSC et al. [11], 14.5% of COPD patients had sarcopenia overall. Maria et al. reported that sarcopenia was common in 24.6% of COPD patients [12]. According to the study by Mahmoud A. Qora et al. [13], normal renal function, concealed CRF, and overt CRF were present in 54%, 26%, and 20% of COPD patients, respectively.

In the present study, the prevalence of sarcopenia is higher than that found in the studies referenced in [10] and [11] when comparing the results of the current study to the previously mentioned studies. However, in comparison to the prevalence reported in the studies mentioned in [9] and [13], the present study indicates a lower prevalence of both overt and concealed CRF. It is crucial to remember that the prevalence may vary depending on the population under study, the terminology used, and other factors. Therefore, these factors should be considered when comparing the findings of different studies.

Age groups greater than 60 years, urban residents, and prolonged hospital stays (>10 days) were found to be statistically significant factors associated with sarcopenia in COPD patients. This result can be contrasted with a study's discovery that, in response to enhanced catabolism, raised proinflammatory cytokines, and increased oxidative stress [12], COPD might be considered a risk factor for sarcopenia. Another study revealed that age, nutritional status, exercise ability, COPD severity, functional performance, smoking status, concomitant disorders, and self-reported hospital admission were all linked to sarcopenia in COPD patients [10]. Furthermore, a systematic review and meta-analysis revealed that the prevalence of sarcopenia in COPD patients, based on current criteria for sarcopenia diagnosis, is low, and the factors associated with sarcopenia in COPD, particularly those related to geriatric syndromes like falling and disability, have not been extensively studied [14].

Previous studies have demonstrated that sarcopenia constitutes a risk factor for adverse outcomes in older individuals. However, the definitive confirmation of this relationship in chronic kidney disease (CKD) remains elusive, as indicated by the current study investigating the association between sarcopenia and chronic renal failure. Moreover, a systematic review and meta-analysis have linked low muscle mass, reduced muscular strength, and impaired physical performance to increased mortality among CKD patients [15]. Notably, among CKD patients, the loss of muscle mass is notably more severe and occurs earlier than anticipated, with younger individuals exhibiting initial signs of sarcopenia [16]. This observation aligns with the findings of a study conducted by Sepúlveda-Loyola et al., which examined the influence of body composition and sarcopenia on mortality in COPD patients [17]. Another study by Wang et al. [18] reviewed the relationship between body composition and COPD, reporting a higher prevalence of sarcopenia in COPD patients compared to age-matched controls. Additionally, research by Costa et al. revealed that sarcopenia was prevalent in 39.6% of COPD patients and was linked to reduced muscle strength and exercise capacity [19]. These collective findings underscore the significance of sarcopenia and its impact on various medical conditions, emphasizing the need for further research and clinical attention to this important aspect of patient health. 

The impact of sarcopenia on pulmonary function tests, such as FEV1, has also been investigated. A study by Jaitovich et al. found that sarcopenia was associated with decreased lung function in COPD patients, as measured by FEV1 [20]. Likewise, a study by Marquis et al. reported that sarcopenia was associated with decreased exercise capacity and increased dyspnea in COPD patients [21]. In addition to its impact on physical function, sarcopenia has also been associated with changes in laboratory investigations in COPD patients. A study by Swallow et al. found that sarcopenia was associated with decreased serum albumin levels in COPD patients, which is a marker of malnutrition [22]. Another study by Engelen et al. reported that sarcopenia was associated with decreased serum creatinine levels in COPD patients, which is a marker of muscle mass [23].

Recommendations

In order to gain a deeper understanding of the relationship between sarcopenia, chronic renal failure (CRF), and COPD, it is advisable to consider the implementation of a longitudinal study design. Such an approach would allow for the tracking of participants over an extended period, offering insights into the causal dynamics of these conditions within COPD patients. Furthermore, a more comprehensive understanding of the mechanisms connecting these conditions could be achieved by incorporating relevant biomarkers, including those related to muscle mass and function, renal function, and inflammation. Lastly, it is essential to explore potential interventions or treatments that could help mitigate the development or progression of sarcopenia and CRF in COPD patients. Such interventions might involve exercise regimens, nutritional interventions, or pharmacological approaches.

Limitations

One notable limitation of the current study lies in its cross-sectional nature, which makes it challenging to establish causal relationships between sarcopenia, CRF, and COPD. It remains uncertain whether sarcopenia leads to CRF, CRF leads to sarcopenia, or if there exists a bidirectional relationship. Additionally, the study's small sample size raises concerns about sampling bias, potentially limiting the generalizability of its findings to the broader COPD population. It's crucial for future research to address this limitation by including a more diverse and representative sample. 

Overall, while there is only some research on the relationship between sarcopenia and COPD and between sarcopenia and chronic renal failure, there is a lack of studies investigating the association between sarcopenia, COPD, and chronic renal failure in COPD patients. The present study provides valuable insights into this relationship and highlights the importance of early detection and management of sarcopenia in COPD patients to optimize their clinical outcomes. Further research is needed to confirm these findings and to better understand the mechanisms underlying the association between sarcopenia, COPD, and chronic renal failure.

## Conclusions

The study provides evidence for the association between sarcopenia and chronic renal failure in COPD patients. The study found that sarcopenia is associated with various negative clinical outcomes, including reduced pulmonary function, increased dyspnea, and lower levels of hemoglobin, GFR, and total protein. The study highlights the importance of early detection and management of sarcopenia and CRF in COPD patients to optimize their clinical outcomes.

## References

[1] Kim SH, Shin MJ, Shin YB, Kim KU (2019). Sarcopenia associated with chronic obstructive pulmonary disease. J Bone Metab.

[2] Zhao X, Su R, Hu R (2023). Sarcopenia index as a predictor of clinical outcomes among older adult patients with acute exacerbation of chronic obstructive pulmonary disease: a cross-sectional study. BMC Geriatr.

[3] Choi YJ, Kim T, Park HJ, Cho JH, Byun MK (2023). Long-term clinical outcomes of patients with chronic obstructive pulmonary disease with sarcopenia. Life (Basel).

[4] Sabatino A, Cuppari L, Stenvinkel P, Lindholm B, Avesani CM (2021). Sarcopenia in chronic kidney disease: what have we learned so far?. J Nephrol.

[5] Demircioğlu H, Cihan FG, Kutlu R, Yosunkaya Ş, Zamani A (2020). Frequency of sarcopenia and associated outcomes in patients with chronic obstructive pulmonary disease. Turk J Med Sci.

[6] Chen LK, Woo J, Assantachai P (2020). Asian Working Group for Sarcopenia: 2019 consensus update on sarcopenia diagnosis and treatment. J Am Med Dir Assoc.

[7] Ertan Yazar E, Niksarlioglu EY, Yigitbas B, Bayraktaroglu M (2022). How to utilize CAT and mMRC scores to assess symptom status of patients with COPD in clinical practice?. Medeni Med J.

[8] Formiga MF, Vital I, Urdaneta G, Balestrini K, Cahalin LP, Campos MA (2018). The BODE index and inspiratory muscle performance in COPD: Clinical findings and implications. SAGE Open Med.

[9] Madouros N, Jarvis S, Saleem A, Koumadoraki E, Sharif S, Khan S (2022). Is there an association between chronic obstructive pulmonary disease and chronic renal failure?. Cureus.

[10] Limpawattana P, Inthasuwan P, Putraveephong S, Boonsawat W, Theerakulpisut D, Sawanyawisuth K (2018). Sarcopenia in chronic obstructive pulmonary disease: A study of prevalence and associated factors in the Southeast Asian population. Chron Respir Dis.

[11] Jones SE, Maddocks M, Kon SS (2015). Sarcopenia in COPD: prevalence, clinical correlates and response to pulmonary rehabilitation. Thorax.

[12] Tsekoura M, Tsepis E, Billis E, Gliatis J (2020). Sarcopenia in patients with chronic obstructive pulmonary disease: A study of prevalence and associated factors in Western Greek population. Lung India.

[13] Elmahallawy II, Qora MA. (2013). Prevalence of chronic renal failure in COPD patients. Egyptian Journal of Chest Diseases and Tuberculosis.

[14] Benz E, Trajanoska K, Lahousse L (2019). Sarcopenia in COPD: a systematic review and meta-analysis. Eur Respir Rev.

[15] Ribeiro HS, Neri SG, Oliveira JS, Bennett PN, Viana JL, Lima RM (2022). Association between sarcopenia and clinical outcomes in chronic kidney disease patients: A systematic review and meta-analysis. Clin Nutr.

[16] Formiga F, Moreno-González R, Corsonello A (2022). Diabetes, sarcopenia and chronic kidney disease; the Screening for CKD among Older People across Europe (SCOPE) study. BMC Geriatr.

[17] Sepúlveda-Loyola W, Osadnik C, Phu S, Morita AA, Duque G, Probst VS (2020). Diagnosis, prevalence, and clinical impact of sarcopenia in COPD: a systematic review and meta-analysis. J Cachexia Sarcopenia Muscle.

[18] Wang X, Liang Q, Li Z, Li F (2023). Body composition and COPD: a new perspective. Int J Chron Obstruct Pulmon Dis.

[19] Costa TM, Costa FM, Moreira CA, Rabelo LM, Boguszewski CL, Borba VZ (2015). Sarcopenia in COPD: relationship with COPD severity and prognosis. J Bras Pneumol.

[20] Jaitovich A, Barreiro E (2018). Skeletal muscle dysfunction in chronic obstructive pulmonary disease. What we know and can do for our patients. Am J Respir Crit Care Med.

[21] Marquis K, Debigaré R, Lacasse Y, LeBlanc P, Jobin J, Carrier G, Maltais F (2002). Midthigh muscle cross-sectional area is a better predictor of mortality than body mass index in patients with chronic obstructive pulmonary disease. Am J Respir Crit Care Med.

[22] Swallow EB, Reyes D, Hopkinson NS (2007). Quadriceps strength predicts mortality in patients with moderate to severe chronic obstructive pulmonary disease. Thorax.

[23] Engelen MP, Schols AM, Does JD, Wouters EF (2000). Skeletal muscle weakness is associated with wasting of extremity fat-free mass but not with airflow obstruction in patients with chronic obstructive pulmonary disease. Am J Clin Nutr.

